# The magnitude of stunting and its determinants among late adolescent girls in East Africa: Multilevel binary logistics regression analysis

**DOI:** 10.1371/journal.pone.0298062

**Published:** 2024-05-09

**Authors:** Hiwot Altaye Asebe, Beminate Lemma Seifu, Kusse Urmale Mare, Bizunesh Fantahun Kase, Tsion Mulat Tebeje, Yordanose Sisay Asgedom, Abdu Hailu Shibeshi, Afewerk Alemu Lombebo, Kebede Gemeda Sabo, Bezawit Melak Fente, Zufan Alamrie Asmare

**Affiliations:** 1 Department of Public Health, College of Medicine and Health Sciences, Samara University, Afar, Ethiopia; 2 Department of Nursing, College of Medicine and Health Sciences, Samara University, Afar, Ethiopia; 3 School of Public Health, College of Health Science and Medicine, Dilla University, Dilla, Ethiopia; 4 Department of Epidemiology and Biostatics, College of Health Sciences and Medicine, Wolaita Sodo University, Soddo, Ethiopia; 5 Department of Statistics, College of Natural and Computational Science, Samara University, Afar, Ethiopia; 6 School of Medicine, College of Health Science and Medicine, Wolaita Sodo University, Soddo, Ethiopia; 7 Department of General Midwifery, School of Midwifery, College of Medicine & Health Sciences, University of Gondar, Gondar, Ethiopia; 8 Department of Ophthalmology, School of Medicine and Health Science, Debre Tabor University, Debre Tabor, Ethiopia; India Health Action Trust (IHAT), Uttar Pradesh Technical Support Unit (UP-TSU), INDIA

## Abstract

**Background:**

Stunting poses a significant health risk to adolescent girls aged 15–19 in low- and middle-income countries, leading to lower education levels, reduced productivity, increased disease vulnerability, and intergenerational malnutrition. Despite the inclusion of adolescent nutrition services in the Sustainable Development Goals, little progress has been made in addressing malnutrition among adolescent girls in several African nations. Limited evidence exists in East Africa due to small sample sizes and methodological limitations. To overcome these constraints, this study utilizes the latest Demographic and Health Survey data to estimate the prevalence and factors influencing stunting among late adolescent girls in ten East African countries.

**Methods:**

This study utilized the most recent Demographic and Health Survey (DHS) data from 10 East African countries, including a total sample weight of 22,504 late-adolescent girls. A multilevel mixed-effect binary logistic regression model with cluster-level random effects was employed to identify factors associated with stunting among these girls. The odds ratio, along with the 95% confidence interval, was calculated to determine individual and community-level factors related to stunting. A p-value less than 0.05 was considered statistically significant in determining the factors influencing stunting among late-adolescent girls.

**Results:**

The prevalence of stunting among late adolescent girls in East Africa was found to be 13.90% (95% CI: 0.13–0.14). Religion, relationship to the head, presence of under-five children in the household, lactating adolescent, marital status, Time to get water source, and country of residence were significantly associated with Stunting.

**Conclusion:**

This study highlights the complexity of stunting in East Africa and identifies key factors that need attention to reduce its prevalence. Interventions should focus on improving water access, supporting lactating girls, addressing socioeconomic disparities, promoting optimal care practices, and implementing country-specific interventions to combat stunting and improve adolescent girls’ nutrition.

## Introduction

Adolescence refers to the transitional phase when a child progresses into adulthood. Late adolescent girls, specifically those aged 15–19, experience this stage [1,2]. Typically, adolescents are expected to be in good health and have a lower susceptibility to infections compared to young children and older adults. Consequently, they often receive limited healthcare and nutritional attention, except for reproductive health concerns [2]. However, due to their increased nutritional requirements for growth, development, and sexual maturation, including the onset of menstruation, adolescent girls are at risk of stunting [3].

These young girls often face harsh environmental conditions and are required to do physically demanding work. This can add an extra layer of physiological stress and increase their nutritional needs during this crucial phase of growth. In certain cultures, the risk is even higher for girls, right from infancy through adolescence, due to biases based on their gender [4,5]. This issue is particularly prevalent among 15- to 19-year-old girls in low- and middle-income countries [2].

Stunting among adolescent girls is a significant global public health issue [6,7]. Policymakers have not given enough attention to the issue, despite its significant impact on late adolescent females in developing nations [2]. The prevalence of stunting among adolescent girls is highest in the South Asian and sub-Saharan African (SSA) regions compared to other regions of the globe [2]. As per a 2018 report by the World Health Organization (WHO), the region of South Asia has the highest prevalence of stunting among adolescents. The report states that in South Asia, approximately 36% of adolescents aged 10–19 years are stunted. This is followed by sub-Saharan Africa, where around 30% of adolescents are stunted. Additionally, a secondary analysis of 11 Asian and SSA countries identified Nigeria as having the highest prevalence of stunting among adolescent girls, with a rate of 32.90% [8]. Likewise, East African nations bear a significant burden of stunting among late-adolescent girls. [2].

For example, according to a report by the World Health Organization (WHO), the country in Africa with the highest prevalence of stunting among adolescents is Ethiopia. The report states that 38.4% of adolescents in Ethiopia suffer from stunted growth. Other African countries with high rates of stunting among adolescents include Madagascar (35.10%), Burundi (29.50%), and Eritrea (27.90%) [9].

In addition, inadequate nutrition leads to negative outcomes in both development and health. These include lower educational attainment, reduced productivity, and an increased susceptibility to communicable and non-communicable illnesses [10]. Research has demonstrated that stunted adolescent girls have a higher probability of continuing to be malnourished as mothers, resulting in the birth of infants with small birth weights. This creates a cycle of malnutrition that spans generations [11,12]. Furthermore, inadequate nutrition during adolescence can contribute to stillbirths, neonates with restricted growth during pregnancy, complicated deliveries, and even maternal mortality [13].

Numerous research studies have provided evidence that factors such as age, level of education, occupation, family size, lack of proper sanitation facilities, dietary diversity, and residential location are closely linked to stunting [14–18].

Although the Sustainable Development Goals (SDGs) include an adolescent nutrition service to tackle malnutrition among adolescents, the nutritional condition of adolescent girls in numerous African nations has not demonstrated any progress[19]. It is crucial to have evidence on the prevalence and determinants of stunting among late adolescent girls at the regional level to monitor the progress of health-related targets of the SDGs. Nonetheless, there is a scarcity of comprehensive data in East African countries regarding the magnitude and underlying factors contributing to stunting among late-adolescent girls. Furthermore, previous studies have relied on small sample sizes and have been conducted in a restricted geographic area and some of these studies do not employ appropriate statistical method (i.e. multilevel analysis) that accounts for the hierarchical nature of data [20]. Thus, considering the previously mentioned constraints, the objective of this study was to utilize the latest Demographic and Health Survey (DHS) data to estimate the occurrence and factors influencing stunting among late adolescent girls in ten East African countries.

## Methods

### Data source, study setting, and population

This study utilized data from the Demographic and Health Surveys (DHS) conducted in ten East African nations from 2011 to 2021, with the countries being selected based on the availability of their DHS data and the required measurements within this data. The DHS is a survey that encompasses a representative sample of the entire population at the national level and is routinely conducted every five years. It collects data on basic health indicators like mortality, morbidity, fertility, and maternal and child health-related characteristics. A two-stage stratified sampling approach was employed to select participants for the survey. During the initial stage, enumeration areas (EAs) were randomly selected based on the country’s recent population, and using the housing census as a sampling frame, households were randomly selected in the second stage. The survey conducted in each country comprised distinct datasets encompassing information on men, women, children, births, and households. Regarding this particular research, the study population was late adolescent girls; thus, we used the individual (women’s) record dataset (IR file). In the current study a weighted sample of 22504, late-adolescent girls was considered for final analysis. Detailed information about DHS methodology can be found in the official database https://dhsprogram.com/Methodology/index.cfm.

### Study variables and definitions

The outcome variable was Stunting among late adolescent girls, which was assessed by height for age < −2 scores of the 2007 WHO reference. For the analysis purpose, adolescents were grouped into two categories of nutritional status based on their height for age z-score: stunted and not stunted. Explanatory variables were classified as individual and community-level factors. Explanatory variables were Socioeconomic, demographic, and Environmental factors were selected based on previous studies and their availability in the DHS dataset [21–25]. Individual level variables considered in this study were age, marital status, educational status, sex of head, number of under-five, current working status, breastfeeding, religion, relationship to head, age of household head, wealth index, source of drinking water, time to get to a water source, and latrine. Place of residence, living country, community literacy level, and community poverty were community-level variables. The last two community-level variables were generated by aggregating the individual-level observations at the cluster level (i.e. they are not directly found in the Demographic Health Survey dataset). The aggregates were computed using the average values of the proportions of women in each category of a given variable and median values were used to categorize the aggregated variables into groups (i.e. low and high).

### Data management and statistical analysis

Data from 10 East African countries were pooled, recoded, and analyzed using Stata version 17 software. The WHO Anthro-plus software was used to form HAZ scores. The data underwent weighting using sampling weight, primary sampling unit, and strata to restore the representativeness of the survey and obtain accurate estimates. Descriptive results were presented using frequencies and percentages.

A multilevel binary logistic regression model was applied to determine the effects of each independent variable. Bivariable multilevel logistic regression analysis was used to identify variables eligible for the multivariable analysis. Variables with a p-value less than 0.25 in this analysis and those found important in the literature were considered candidates for multilevel binary logistic regression analysis.

#### Model building

The likelihood ratio (LR) test, intra-class correlation coefficient (ICC), and median odds ratio (MOR) were computed to measure the variation between clusters. Intra-class correlation coefficients (ICC) were computed to measure the variation between clusters. Data eligibility for multilevel analysis was checked before analysis (intra-class correlation coefficient (ICC) greater than 10% (ICC = 2.38%). Hence the ICC<10%. Even though the ICC value was less than 10%, the log-likelihood ratio (LR) was significant, indicating that a multilevel binary logistic regression model better fits the data than the classical regressions. The log-likelihood ratio test (X2 = 24.90, p-value < 0.001) informed us to choose a mixed-effect logistic regression model over the basic model [26].

$$ICC={ϭ}^{2}/({ϭ}^{2}+\pi^{2}/3).$$

The MOR quantifies the variation or heterogeneity in malnutrition between clusters in terms of the odds ratio scale and is defined as the median value of the odds ratio between the cluster with a high likelihood of malnutrition and the cluster at lower risk when randomly picking out individuals from two clusters (EAs) [27]

$$MOR=exp\sqrt{\left(2*\partial 2*0.6745\right)}∼MOR=exp(0.95*\partial ).$$

∂^2^ indicates the cluster variance

After selecting the variables for multivariable multilevel binary logistic regression analysis, four models were fitted to identify the best-fitted model. Model-I or null model (a model with only outcome variable), model-II (a model with only individual-level explanatory variables), model-III (a model with only community-level explanatory variables), and model-IV/full model (simultaneously examined the effect of both individual and community-level predictors on the outcome variable) were fitted. The models were compared with deviance (-2Log-likelihood Ratio (-2LLR)) and the mixed effect binary logistic regression model best fitted the data since it had the lowest deviance value.

Statistical significance of the independent variables was determined using a p-value < 0.05 and adjusted odds ratio along with a corresponding 95% Confidence Interval (CI). Collinearity diagnostic was performed, and the Variance Inflation Factor (VIF) values for all variables in the final model were below 10, indicating the absence of multicollinearity.

### Ethical consideration

This research did not necessitate ethical approval or participant consent as it involved analyzing existing survey data publicly accessible through the MEASURE DHS program. We have received authorization to acquire and utilize the data from https://www.dhsprogram.com/data/dataset_admin/login_main.cfm for our study. The datasets do not contain any personal information such as names or addresses of individuals or households.

## Patient and public involvement statement

This secondary data analysis was not designed or planned with patients or the general public in mind. However, it is critical for the initial data collection process because household measurements such as height were collected to calculate stunting.

## Result

This study included 22,504 adolescent girls between the ages of 15 and 19, who participated in the DHS survey conducted in various countries. In East African countries, the majority of the girls (63.30%) lived in rural areas. Concerning the economic status of the families these girls belonged to, 34.79% had a low wealth index. Additionally, in East African countries, 38.55% of households took 30 minutes or more to reach the water source (Table 1).

**Table 1:** Sociodemographic and economic characteristics of 10 East African countries from recent DHS data.

Variables	Categories	Frequency (%)
Age of respondents	15–17	13,708 (60.91)
	18-19	8,796 (39.09)
Residence	Urban	6,907 (30.69)
	Rural	15,597(69.31)
Current marital status	Never in union	17,362(77.15)
	Married	3,376 (15)
	Live with partner	1,151(5.12)
	Divorced	599 (2.66)
	Widowed	14(0.06)
Sexof house-hold head	Female	6,931(30.80)
	Male	15,573(69.20)
Age of head (continuous)		22504(100)
Number of under-five children	No	9,416(41.84)
	At least one child	13,088(58.16)
Family wealth index	Poorest	3,741(16.62)
	Poorer	4,088(18.16)
	Middle	4,355(19.35)
	Richer	4,610(20.49)
	Richest	5,710(25.37)
Educational status	No formal education	1,614(7.17)
	Formal education	20,889(92.83)
Religion	Orthodox	1,296(6.54)
	Catholic	4,670(23.57)
	Protestant	9,267(46.77)
	Muslim	3,220(16.25)
	Traditional	534(2.69)
	Other	825(4.16)
Relationship to the household head	Head	667(2.96)
	Wife	2,834(12.60)
	Daughter	12,217(54.29)
	daughter-in-law	802(3.56)
	Granddaughter	1,588(7.06)
	sister	672(2.99)
	other relatives	2,105(9.35)
	adopted/foster child	570(2.53)
	Not related	1,048(4.66)
Occupation	Not working	13,747(61.09)
	Office	228(1.01)
	Sales	1,366(6.07)
	Agriculture	5,253(23.34)
	Domestic	623(2.77)
	Manual	1,159(5.15)
	Other	127(0.56)
Sources of drinking water	Unprotected	6,320(28.09)
	Protected	16,184(71.91)
Type of toilet	Not improved	14,078(62.56)
	Improved	8,426(37.44)
Media exposure	Yes	12,075(53.65)
	No	10,429(46.35%)
Living Country	Comoros	1,164(5.17)
	Burundi	1,747(7.76)
	Ethiopia	3,100(13.77)
	Kenya	2,501(11.11)
	Madagascar	2,017(8.96)
	Malawi	1,699(7.55)
	Mozambique	2,885(12.82)
	Tanzania	2,681(11.91)
	Uganda	1,291(5.74)
	Zambia	3,420(15.20)

### The prevalence of stunting among late adolescent girls in East Africa

The prevalence of stunting among late adolescent girls in East Africa was 13.90% (95% CI; (0.13- 0.14), Zambia 16.40(95% CI: 0.15- 0.18) and Uganda 5.70%(95% CI: 0.049 – 0.066) had the highest and lowest prevalence respectively (Table 2).

**Table 2:** Frequency distribution of stunting in East Africa.

Country	Year of survey	Total frequency	Stunting Yes (%)
Comoros	2012	1,164(5.17)	187(16.10)
Burundi	2016/17	1,747(7.76)	260(14.90)
Ethiopia	2016	3,100(13.77)	433.(14.00)
Kenya	2014	2,501(11.11)	247(9.90)
Madagascar	2021	2,017(8.96)	279(13.80)
Malawi	2015/16	1,699(7.55)	214(12.60)
Mozambique	2011	2,885(12.82)	441(14.20)
Tanzania	2015/16	2,681(11.91)	378(14.10)
Uganda	2016	1,291(5.74)	179(13.90)
Zambia	2013/14	3,420(15.20)	179(5.20)

### Statistical analysis and model comparison

The MOR for malnutrition was 1.14 in the null model which indicates that there was variation between clusters. If we randomly select an individual from two different clusters, individuals at the cluster with a higher risk of malnutrition had 1.14 times higher odds of being malnourished as compared with individuals at the cluster with a lower risk of malnutrition. The models were compared with deviance and model IV was chosen as the best-fitted model since it had the lowest deviance value (18188) Table 3.

**Table 3:** A measure of variation for stunting among adolescent girls at cluster level by multilevel binary regression analysis from most recent DHS data.

Measure of variation	Null model	Model 1	Model 2	Model3
MOR	1.14	1.14	1.139	1.138
ICC (%)	2.40	2.28	2.12	2.06
Model fitness	Null model	Model 1	Model 2	Model3
Deviance	18320.33	18270.53	18239.30	18188
Mean VIF				2.50

### Determinants of stunting among late adolescent girls in East Africa

The results from the multivariable model reveal that Religion, relationship to head, presence of under-five children in the household, lactating adolescent, marital status Time to get water source, and country of residence were significantly associated with Stunting in East Africa. This study revealed that the odds of stunting were 3.13 (AOR =3.13; 95% CI: 1.04-9.45) times higher among widowed adolescent girls as compared to those married. Adolescent girls who walked 30 minutes and above to fetch water had a 1.10 times higher risk of stunting (AOR =1.10; 95% CI: 1.007-1.19). Adolescents who are followers of the Orthodox religion have1.43times (AOR =1.43; 95% CI: 1.13-1.82) higher risk of being stunted than those who are followers of the protestant religion. The odds of stunting among non-lactating late adolescent girls reduced by 13% (AOR =0.87; 95% CI: 0.76-0.99). Moreover, for those adolescent girls found in households that had at least one under-five child, the odds of stunting decreased by 12%, compared to adolescent girls living in households that had no under-five child. Regarding to relationship to household head, those who are not related by blood to the head had 1.50 times (AOR = 1.5; 95% CI: 1.09- 2.1) higher risk stunting as compared to the head. The odds of stunting among granddaughters of the head is 1.50 (AOR = 1.50; 95% CI: 1.05- 2.11) times higher than the head of household. Livings in Zambia have 1.92 times (AOR = 1.64; 95% CI: (1.61-2.27) higher risk of being stunted as compared to living in Kenya (see Table 4).

**Table 4:** Determinants of stunting among late adolescent girls in East African countries from most recent DHS data.

Variables	Categories	Stunting		Model I AOR(95%CI)	Model II AOR(95%CI)	Model III AOR(95%CI)
		Stunted	Not stunted			
marital status	Never in union	2,397	14,965	1.06(0.87-1.3)		1.08(0.88-1.32)
	Married	488	2,889	1		1
	Live with partner	170	981	1.10 (0.9-1.34)		1.06(0.87-1.29)
	Divorced	74	525	1.01(0.74- 1.37)		1.00(0.73-1.36)
	Widowed	3	12	3.12(1.03-9.41)		3.13(1.04-9.45)*
Religion	Orthodox	187	1,109	1.18(0.99- 1.39)		1.43(1.13-1.82)*
	Catholic	716	3,954	1.09(0.98-1.20)		1.10(0.96-1.20)
	Protestant	1,178	8,089	1		1
	Muslim	477	2,743	1.13(1.01- 1.26)		1.20(1.02- 1.42)*
	Traditional	85	448	1.2(0.95-1.50)		1.10(0.85-1.41)
	Other	110	715	0.97(0.79-1.20)		0.90(0.72-1.13)
Age of head				0.99(0.99-1.00)		0.99(0.99-1.00)
Number of under-five children	No	1,362	8,053	1		
	At least one child	1,769	11,319	0.91(0.83-0.98)		0.88(0.81-0.95)*
Family wealth index	Poorest	571	3,170	1		1
	Poorer	570	3,517	0.99(0.88-1.14)		0.98(0.86-1.12)
	Middle	568	3,787	0.96(0.84-1.09)		0.93(0.8- 1.06)
	Richer	644	3,966	1.01(0.89-1.16)		0.96(0.84-1.11)
	Richest	778	4,931	0.97(0.84- 1.11)		0.91(0.79- 1.06)
Educational status	No formal education	273	1,341	1.08(0.9-1.26)		1.09(0.93-1.28)
	Formal education	2,858	18,030	1		1
Breastfeeding	No	2,689	16,766	0.87(0.76-0 .99)		0.87(0.76-0.99)*
	Yes	443	2,606	1		1
Relationship to the household head	Head	78	589	1		1
	Wife	422	2,412	1.17(0.89-1.54)		1.17(0.89-1.54)
	Daughter	1,619	10,598	1.25(0.94-1.66)		1.25(0.94-1.66)
	daughter-in-law	100	702	1.26(0.88-1.79)		1.26(0.89-1.80)
	Granddaughter	243	1,345	1.49(1.06-2.11)		1.50(1.05- 2.11)*
	sister	97	575	1.40(1.01-1.94)		1.37(0.99-1.90)
	other relatives	318	1,787	1.29(0.96-1.72)		1.25(0.94-1.68)
	adopted/foster child	88	481	1.44(1.01-2.05)		1.37(0.95-1.95)
	Not related	166	882	1.54(1.12-2.14)		1.50(1.09- 2.1)*
Occupation	Not working	1,836	11,912	1		1
	Office	24	204	0.83(0.54-1.28)		0.85(0.55-1.32)
	Sales	203	1,163	1.02(0.87- 1.20)		1.03(0.87-1.21)
	Agriculture	778	4,475	1.13(1.02-1.24)		1.06(0.96-1.18)
	Domestic	85	537	0.85(0.65- 1.12)		0.89(0.67-1.19)
	Manual	177	981	1.13(0.96- 1.34)		1.09(0.92-1.31)
	Other	26	100	1.41(0.83-2.41)		1.45(0.85- 2.48)
Time to get a water source	30 min and above	1,111	6,680	0.93(0.86-1.02)		1.10(1.007-1.19)*
	Less than 30 min	2,021	12,692	1		1
Community poverty	High	1,635	9,831		1	1
	Low	1,497	9,541		1.01(0.9-1.13)	0.98(0.89-1.08)
Community Literacy	High	2,205	14,039		1.04(0.96-1.13)	1.01(0.90-1.13)
	Low	926	| 5,333		1	1
Living Country	Comoros	187	977		2.01(1.6-2.48)	1.80(1.41-2.32)*
	Burundi	260	1,487		1.75(1.45-2.12)	1.70(1.39-2.10)*
	Ethiopia	433	2,667		1.65(1.39- 1.97)	1.35(1.09-1.69)*
	Kenya	247	2,254		1	1
	Madagascar	279	1,738		1.79(1.49-2.16)	1.83(1.512.23)*
	Malawi	214	1,485		1.46(1.19-1.78)	1.46(1.19-1.79)*
	Mozambique	441	2,444		1.80(1.52- 2.15)	1.83(1.52-2.21)*
	Tanzania	378	2,303		1.80(1.47- 2.14)	1.86(1.52-2.27)*
	Uganda	179	1,111		1.73(1.4-2.12)	1.65(1.33-2.05)*
	Zambia	514	2,906		1.80(1.53-2.13)	1.92(1.61-2.27)*

Key

* implies significance at p-value < 0.05 and adjusted odds ratio (AOR): Holds other relevant variables constant and provides the odds ratio for the potential variable of interest which is adjusted for the other independent variables included in the model.

## Discussion

This research examined the prevalence of stunting among late adolescent girls in 10 East African countries. The overall magnitude of stunting was found to be 13.90% (95% CI; (0.13- 0.14), Zambia 16.40(95% CI: 0.15- 0.18), and Uganda 5.70%(95% CI: 0.049 – 0.066) had the highest and lowest prevalence respectively. Religion, relationship to head, presence of under-five children in the household, distance to water source, marital status, lactating adolescent, and country of residence were significantly associated with Stunting in East Africa.

According to a study conducted by the World Health Organization (WHO) in 2018, the overall prevalence of stunting among adolescent girls aged 10-19 years in low- and middle-income countries was estimated to be 13.80%. This is very similar to the prevalence found among late adolescent girls in East Africa, which was reported to be 13.90% [9].

However, it is important to note that stunting prevalence varies widely across different regions and countries. For example, a study conducted in India found that the prevalence of stunting among adolescent girls was as high as 34.40% [28]. Another study conducted in Pakistan reported a prevalence of 32.30% among adolescent girls [29]. On the other hand, some studies have reported lower prevalence rates of stunting among adolescent girls. A study conducted in Indonesia found a prevalence of 25%, while another study in Brazil reported a prevalence of 7.80% [30,31]. The variation in stunting prevalence among adolescent girls can be attributed to several factors such as socio-economic status, access to healthcare and nutrition, and cultural practices. Policymakers and healthcare providers need to consider these factors when developing interventions aimed at reducing the burden of stunting among adolescent girls.

This study revealed that being a follower of Orthodox and Muslim religions increases stunting as compared to those who are protestant. This finding is in line with the study conducted in India and Ethiopia [32,33]. This difference could be attributed to dietary restrictions imposed by Orthodox Christianity, such as fasting periods that may limit nutrient intake during critical growth periods.

Adolescent girls who walk for 30 minutes and above to fetch water had a higher risk of stunting as compared to those who walk for less than 30 min. This finding is in line with the study conducted in India [34]. This might be due to it demanding physical strength and consuming a significant portion of their day. This could lead to changes in their eating patterns. Moreover, the time they spend on this task is time taken away from other important activities, such as their education or rest [35].

This study revealed that the odds of stunting were 3.13 (AOR =3.13; 95% CI: 1.04-9.45) times higher among adolescent girls who were widowed. This could be attributed to various factors such as adolescent girls experiencing widowhood may undergo substantial stress, marked by increased cortisol levels. Chronic stress has been linked to alterations in hormonal regulation, potentially affecting the production of growth hormones in the body. The cascade of stress-induced hormonal changes, including elevated cortisol, may contribute to a physiological environment that hampers optimal linear growth [36,37].

Moreover, the impact of widowhood extends beyond stress to encompass alterations in lifestyle and nutrition patterns. Changes in dietary habits sleep disruptions, and other lifestyle adjustments may further compound the risk of stunting. These lifestyle factors, when combined with a genetic predisposition that influences metabolic processes or hormonal regulation, could contribute synergistically to the observed association [38].

The risk of stunting among non-lactating adolescent girls decreased by 13% (AOR =0.87; 95% CI: (0.76-0.98). This finding is in line with a study conducted in Bangladesh the odds of stunting were higher among lactating adolescent girls than non-lactating adolescent girls [39].

In contrast, a study conducted in Vietnam found that there was no significant difference in the prevalence of stunting between lactating and non-lactating adolescent girls [40]. This might be because lactating adolescent girls may face a higher risk of stunting compared to their non-lactating counterparts due to the increased nutritional demands associated with both pregnancy and lactation. During adolescence, girls are still growing and developing, and the concurrent demands of lactation place an additional strain on their nutritional status. The nutritional needs for breastfeeding include an increased intake of energy, protein, vitamins, and minerals, which, if not adequately met, can lead to nutrient deficiencies. Insufficient nutrient intake, especially during this critical period of growth, may result in stunting, characterized by impaired linear growth and development [41,42]. Additionally, lactating adolescent girls may be more vulnerable to socio-economic factors, limited access to nutritious foods, and inadequate healthcare, further exacerbating the risk of stunting. Overall, the relationship between lactation and stunting among adolescent girls appears to be complex and may vary depending on factors such as geographic location, socioeconomic status, and other demographic factors.

Adolescent girls found in households that had at least one under-five child regardless of whether she is the mother of a child or not, the odds of stunting decreased by 12%, compared to adolescent girls living in households that had no under-five child. This might be due to Homes with under-five children having heightened nutritional awareness, leading to better dietary practices for the entire household, including adolescent girls. The presence of young children could emphasize the importance of proper nutrition, positively impacting the overall food environment.

Those who have no blood relationship to the family had 1.50 times (AOR = 1.50; 95% CI: 1.09- 2.1) higher risk of stunting. One study was conducted to examine the association between household structure and child nutritional status in rural Ethiopia and South Africa. The researchers found that children living in households where they were not biologically related to the household head had a higher risk of stunting compared to those who were biologically related [43,44]. This finding aligns with the aforementioned study, suggesting that there is a consistent pattern across different populations.

However, the study explored the relationship between household structure and child nutritional status in rural Bangladesh and did not find a significant association between blood relationship to the household head and stunting risk [45]. This discrepancy could be attributed to differences in cultural contexts, socioeconomic factors, or sample characteristics.

Granddaughters had a 1.5 times higher risk of being stunted. This might be due to some societies; granddaughters may be expected to take on domestic responsibilities at an early age, which could limit their access to education and healthcare. This lack of access to essential services can increase their vulnerability to stunting [46].

### Strengths and limitations of this study

- We analyzed extensive national datasets to identify relevant comparisons for this research.
- The findings of this study are constrained by the utilization of cross-sectional data, which makes it challenging to establish a cause-and-effect relationship between the independent variables and the outcome measures.
- Information regarding well-known factors associated with stunting, such as dietary intake and lifestyle, was included in the data analysis.

## Conclusion

In conclusion, this study highlights the multifaceted nature of stunting in East Africa. It underscores the importance of addressing marital status, lactating status, time to get water source, the presence of under-five children, relationship to household head, and country of residence in interventions aimed at reducing stunting prevalence. Efforts should focus on improving distance to water sources, providing targeted support for lactating girls, addressing socioeconomic disparities, promoting optimal care practices within households, and implementing context-specific interventions in different countries.

## Supporting information

S1 Table This table shows the VIF of variables. (DOCX)

## Abbreviations

AOR: adjusted odds ratio
LLR: Log-likelihood rati
LR: Likelihood ratio
WHO: World Health Organizations
DHS: Demographic Health Survey
EAs: Enumeration Areas
EDHS: Ethiopian Demographic and Health Survey
ICC: Intra-class Correlation Coefficient
HAZ: height for age Z-score
