# Supplementary material for: The magnitude of stunting and its determinants among late adolescent girls in East Africa: Multilevel binary logistics regression analysis
Source: PLoS One. 2024 May 9;19(5):e0298062. doi: 10.1371/journal.pone.0298062 (PMC11081321; doi:10.1371/journal.pone.0298062)
Supplement: S1 Table — (DOCX) [file pone.0298062.s001.docx]

| Variable | VIF | 1/VIF |
| --- | --- | --- |
| Marital status |  |  |
| never in union \| | 5.44 | 0.183837 |
| living with partner | 1.34 | 0.748518 |
| widowed | 1.02 | 0.981603 |
| divorced | 1.64 | 0.609030 |
| Age of head | 2.47 | 0.404633 |
| Number of under five children in the house |  |  |
| At least one child | 1.24 | 0.806146 |
| wealth |  |  |
| poorer | 1.75 | 0.570184 |
| medium | 2.15 | 0.464876 |
| richer | 2.58 | 0.387297 |
| richest | 3.64 | 0.274407 |
| Educational status |  | |
| No formal education | 1.47 | 0.680505 |
| Relation to head |  |  |
| wife | 5.80 | 0.172468 |
| daughter | 12.90 | 0.077541 |
| daughter-in-law | 2.93 | 0.341618 |
| granddaughter | 5.28 | 0.189241 |
| Sister | 2.12 | 0.470607 |
| other relative | 4.86 | 0.205844 |
| adopted/foster child | 2.17 | 0.460676 |
| not related \| | 3.20 | 0.312602 |
| occupation |  |  |
| office | 1.05 | 0.954460 |
| sales | 1.15 | 0.872512 |
| Agriculture | 1.39 | 0.718974 |
| domestic | 1.49 | 0.670758 |
| manual | 1.13 | 0.882236 |
| other | 1.13 | 0.881198 |
| Time to fetch water |  | |
| >30 minute | 1.18 | 0.849296 |
| Community poverty |  | |
| low | 2.19 | 0.456100 |
| Community literacy |  |  |
| high | 1.62 | 0.618683 |
| countries |  |  |
| Burundi | 2.00 | 0.499635 |
| Ethiopia | 2.94 | 0.339810 |
| Comoros | 2.17 | 0.460138 |
| Madagascar | 1.99 | 0.503613 |
| Malawi | 1.70 | 0.587897 |
| Mozambique | 2.37 | 0.421118 |
| Tanzania | 3.65 | 0.273908 |
| Uganda | 1.56 | 0.640342 |
| Zambia | 2.14 | 0.466401 |
| Religion |  |  |
| Orthodox | 2.50 | 0.399674 |
| Catholic | 1.58 | 0.633790 |
| Muslim | 2.17 | 0.460540 |
| Traditional | 1.65 | 0.607347 |
| other | 1.27 | 0.785355 |
| Breast feeding |  |  |
| no | 1.50 | 0.668752 |
| Mean VIF | 2.50 |  |
